# Observer-Related Versus Cross-Modality Variability in Ventricular Quantification by Cardiac CT and Cardiovascular Magnetic Resonance

**DOI:** 10.3390/diagnostics16203280

**Published:** 2026-10-09

**Authors:** Răzvan-Andrei Licu, Alexandra-Mihaela Țolescu-Dobra, Gheorghe-Marian Rotariu, Kincső-Zsófia Lőrincz, Claudia-Raluca Mariean, Marian Pop, Dragoș Constantin Cucoranu, Liviu Moraru, Andrei Manea, Dariana-Elena Pătrîntașu, Anca Dumitrașcu, Anca Bacârea

**Affiliations:** 1 Doctoral School of Medicine and Pharmacy, George Emil Palade University of Medicine, Pharmacy, Science and Technology of Târgu Mureș, 540142 Targu Mures, Romania; 2 Department of Radiology, George Emil Palade University of Medicine, Pharmacy, Science and Technology of Târgu Mureș, 540142 Targu Mures, Romania; 3 Department of Pathophysiology, George Emil Palade University of Medicine, Pharmacy, Science and Technology of Târgu Mureș, 540142 Targu Mures, Romania; 4 Radiology and Medical Imaging Laboratory, Mureș County Emergency Clinical Hospital, 540136 Targu Mures, Romania; 5 Department of Anatomy, George Emil Palade University of Medicine, Pharmacy, Science and Technology of Târgu Mureș, 540142 Targu Mures, Romania; 6 Emergency Institute for Cardiovascular Diseases and Transplantation of Târgu Mureș, 540136 Targu Mures, Romania; 7 Pneumology Department, Mureș Clinical County Hospital, 540142 Targu Mures, Romania

**Keywords:** cardiac computed tomography, cardiovascular magnetic resonance, measurement variability, interobserver reproducibility, repeatability, quantitative imaging

## Abstract

**Background/Objectives**: Ventricular measurements from cardiac computed tomography (CCT) and cardiovascular magnetic resonance (CMR) may differ due to modality-specific, physiological, or observer-dependent reasons. We compared cross-modality disagreement with intra-modality interobserver variability. **Methods**: Fifty-four patients with CCT and CMR examinations within 36 months were retrospectively analyzed. Left ventricular (LV) end-diastolic volume (EDV), right ventricular (RV) EDV, and LV mass were measured on the same platform; 18 underwent independent second-observer measurements. The primary metric was the standard deviation of paired differences [SD(D)], with bootstrap 95% confidence intervals (CIs). **Results**: In the repeat-measurement subset, cross-modality SDs were 29.78 mL, 29.19 mL, and 16.72 g for LV EDV, RV EDV, and LV mass, versus 4.83, 7.33, and 6.71 within CCT and 3.57, 3.58, and 4.11 within CMR. Cross-modality variability was 6.16, 3.98, and 2.49 times the CCT and 8.34, 8.15, and 4.07 times the CMR interobserver variability; all 95% CIs exceeded 1. Examination-interval restriction did not materially reduce dispersion, and body-surface-area indexation changed scale but not relative disagreement. **Conclusions**: Under routine clinical conditions, cross-modality variability in EDV and LV mass exceeded interobserver variability by 2.5- to 8.3-fold. CCT-CMR differences are unlikely to reflect observer-related post-processing variability alone, and consistent modality selection appears preferable to interchanging quantitative thresholds.

## 1. Introduction

Quantitative imaging measurements increasingly influence longitudinal cardiovascular assessment. For a measurement to be clinically useful across time, its technical performance must be understood in terms of bias, repeatability, reproducibility, and the effects of changing measurement conditions [1,2,3]. Method-comparison studies address a related but distinct question: whether two methods can be used with sufficiently similar numerical behavior for the intended clinical purpose. The Bland–Altman methodology emphasizes the distribution of paired differences and the repeatability of each method rather than correlation alone [4]. Reliability and absolute agreement are also conceptually distinct; a high reliability coefficient can coexist with a clinically relevant measurement error when between-subject variability is large [5,6].

Ventricular quantification is particularly suitable for this framework because both cardiovascular magnetic resonance (CMR) and cardiac computed tomography (CCT) can generate chamber volumes and myocardial mass. CMR acquisition and post-processing are extensively standardized [7,8], but body-size normalization [9], contour strategy and observer experience [10,11], and the cine acquisition technique [12] can still influence measured values. Interstudy CMR reproducibility is generally high for both left ventricular (LV) and right ventricular (RV) measurements [13,14], and CCT myocardial mass measurements can also show high reproducibility under consistent analysis conditions [15]. These observations define the ordinary within-modality measurement variation against which cross-modality disagreement should be interpreted.

Prior CCT-CMR studies usually report population-level bias, correlation, concordance, or limits of agreement (LoA). Meta-analytic data show a small, pooled bias between CCT and CMR for LV volumes [16], whereas direct comparison shows non-negligible patient-level dispersion for LV mass [17]. Agreement between CCT and CMR has also been reported for biventricular measurements in postoperative congenital heart disease [18], for RV measurements in a meta-analysis [19], for fully automated analysis of non-contrast chest computed tomography [20], and for LV ejection fraction (EF) in patients after SARS-CoV-2 infection [21]. More recent work has shown that CCT ventricular volumes measured in the mid-diastole are on average lower than those measured at the end-diastole, although the two agree well [22], that the direction and magnitude of CCT-CMR differences depend on the ventricular parameter [23], and that the magnitude of these differences varies with cardiac phase [24]; meta-analytic subgroup data indicate that the contrast protocol, volumetry method, and segmentation approach also affect agreement [19]. In congenital heart disease, the segmentation technique alone changes CCT-derived ventricular volumes [25], and CCT volumetry is used for serial functional follow-up after tetralogy of Fallot repair [26]. However, most studies do not make a clinically useful comparison between cross-modality dispersion and the interobserver variability that occurs when the same modality is remeasured.

CCT and CMR are complementary tools selected according to the clinical question. Their respective roles are defined in modality-specific guidance documents [27,28,29], and appropriate-use criteria address modality selection in congenital and in non-valvular heart disease [30,31]. CCT is valuable for complex congenital and aortic anatomy [32,33], whereas multimodality pathways integrate anatomical, functional, and tissue-characterization information [34,35]. Guideline-directed management also relies on volumetric criteria derived predominantly from CMR: in patients with repaired tetralogy of Fallot, for example, CMR is recommended for serial assessment of RV volumes when pulmonary valve replacement is considered, although the optimal volumetric threshold remains undefined [27]. Commonly applied criteria, such as an indexed RV end-diastolic volume (EDV) of ≥160 mL/m^2^, were derived from CMR, and applying them to CCT measurements may shift intervention decisions [23]. This makes it clinically relevant to determine whether a CCT-CMR difference is comparable to ordinary interobserver variation or if it is substantially larger, because this determines whether CMR-derived thresholds can be applied to CCT measurements without uncertainty beyond that introduced by a change in observer.

The primary objective of this study was therefore to compare the magnitude of cross-modality CCT-CMR disagreement with interobserver variability measured separately within CCT and CMR for LV EDV, RV EDV, and LV myocardial mass. Secondary objectives were to characterize cross-modality variability in the full heterogeneous pediatric–adult cohort and to explore whether examination interval, body surface area (BSA) indexation, measurement magnitude, relative scaling, or age group materially altered disagreement.

## 2. Materials and Methods

### 2.1. Study Design and Population

All patients who underwent clinically indicated CCT and CMR examinations at three collaborating institutions in Târgu Mureș, Romania, between 2016 and 2026 were identified. For the final analytic cohort, the two examinations were required to be separated by no more than 36 months. After application of this temporal criterion, cross-source reconciliation, and quality control, 54 patients had complete paired LV EDV, RV EDV, and LV mass measurements. The cohort included pediatric and adult patients and was not restricted to congenital heart disease. Diagnostic labels were incomplete and were therefore not used for disease-specific inferential analyses. Detailed examination indications were likewise not consistently documented in the available clinical records and could not be retrieved for the complete cohort, so no indication-specific stratification was performed. These records were reviewed for changes in patient management between the paired CCT and CMR examinations. No major changes in medical or surgical management were documented during this interval. No a priori sample-size calculation was performed; all eligible patients satisfying the final criteria were retained. No patient was excluded because the observed CCT-CMR difference was statistically large.

### 2.2. CCT and CMR Acquisition

Imaging was obtained on the clinical systems in routine use at the participating centers over the study period: two GE HealthCare platforms (Revolution Frontier and Revolution HD; GE HealthCare, Chicago, IL, USA) and one Siemens platform (SOMATOM Definition AS; Siemens Healthineers, Forchheim, Germany). All CCT studies were acquired using retrospective electrocardiographic (ECG) gating. No fixed exposure protocol was applied: kilovoltage and milliampere-seconds were chosen case by case according to body habitus, with a gantry rotation time of approximately 0.3 s. Reconstruction differed by vendor, with the GE series generated at a 1.25 mm slice thickness with a 0.625 mm increment and the Siemens series generated at a 0.6 mm increment. Enhancement was achieved with intravenous iodinated agents; the particular agent changed over the decade spanned by the cohort in line with institutional supply, and both injected volume and flow rate were scaled to patient size and to the clinical question. Individualization of this kind is standard in pediatric and congenital CCT [29]. For the present analysis it is a defining condition rather than a shortcoming, since the aim is to quantify disagreement encountered in routine clinical practice rather than under a harmonized research protocol. Multiple cardiac phases were reconstructed from the retrospectively ECG-gated CCT acquisitions. The end-diastolic phase was selected by reviewing the available reconstructed phases and identifying the phase in which the ventricular cavity was largest, following the same phase-selection criterion used for CMR. The end-diastolic phase was thus defined by this image-based criterion and was not assigned to a fixed position relative to the ECG R or Q wave; alternative R-wave- and Q-wave-referenced reconstructions were not available. The analyses used the reconstructed image series archived with each examination. The set of archived phases differed between examinations; the increment between reconstructed phases was not consistently recorded in these series; and the CCT raw data were no longer available. A uniform number and increment of reconstructed phases therefore cannot be reported.

All CMR studies were acquired at 1.5 T, on either an Optima MR450w or a SIGNA Explorer scanner (GE HealthCare, Chicago, IL, USA). Ventricular volumes were derived from a short-axis stack of cine balanced steady-state free-precession images extending from the atrioventricular valve plane through the apex, so that both ventricles were fully encompassed. Sections were 8 mm thick, separated by a 2 mm gap, and each cardiac cycle was resolved into 30 phases. The end-diastolic frame was taken as the phase at which the ventricular cavity reached its largest volume and cross-checked against long-axis cines, in keeping with accepted CMR post-processing convention [7]. Heart rate and blood pressure during the CCT and CMR examinations and ECG trigger timing were not consistently available in the retrospective source data and are therefore not reported.

### 2.3. Image Analysis, Variables, and Indexation

**Figure 1:**
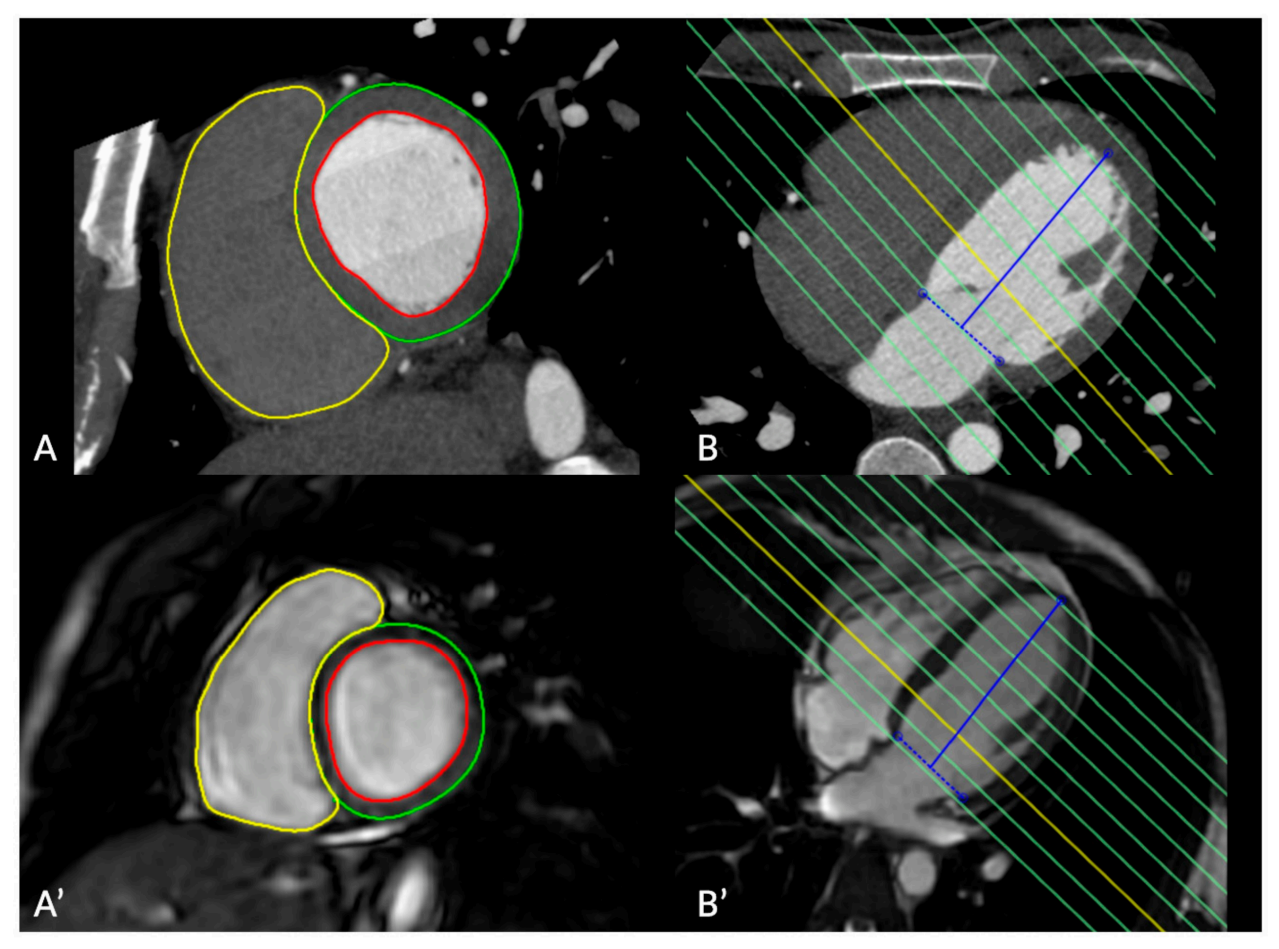
Representative paired CCT and CMR segmentation in the same patient. The upper row shows the CCT examination, and the lower row shows the corresponding CMR examination. Panels (**A**,**A′**) show matched ventricular short-axis segmentation, with the RV endocardial border in yellow, the LV endocardial border in red, and the LV epicardial border in green. Panels (**B**,**B′**) show short-axis stack positioning and basal-plane alignment on orthogonal long-axis views; the solid blue line indicates the LV long axis, from the apex to the center of the mitral valve, and the dashed blue line indicates the mitral valve plane. CCT, cardiac computed tomography; CMR, cardiovascular magnetic resonance; LV, left ventricle; RV, right ventricle.

Both modalities were analyzed on a single post-processing platform (cvi42, version 5.17.2, build 3708; Circle Cardiovascular Imaging, Calgary, AB, Canada), so that measurement conditions differed only in the underlying images. CCT volumes were reformatted into a ventricular short-axis stack oriented perpendicular to the LV long axis, which was defined from the apex to the center of the mitral valve on the four-chamber long-axis reformat. The CCT short-axis stack was generated with an 8 mm section thickness and a 2 mm intersection gap, matching the CMR cine short-axis geometry, so that both modalities were segmented in the same geometric plane and with the same through-plane sampling before any contour was drawn. Contours were generated semi-automatically and manually corrected wherever the automatic result was unsatisfactory. On consecutive short-axis sections, the observer traced the LV endocardium and epicardium and the RV endocardium, applying identical rules to CCT and CMR. Papillary muscles and trabecular tissue were counted as cavity rather than as myocardium and were therefore absent from the LV mass figure. The basal limit of the stack, at the atrioventricular valve planes, and its apical limit were both decided against the corresponding long-axis images, chiefly the four-chamber view. These conventions follow established post-processing principles [7] and standardized cardiac plane orientation and segment nomenclature [36]. Primary measurements were performed by a radiologist with more than 7 years of experience in cardiovascular imaging. A second radiologist with more than 3 years of experience independently repeated the complete analysis for a subset of 18 patients (1/3 of the cohort), randomly selected from the full cohort of 54 subjects, using the same platform and protocol. As for the full cohort, no a priori power calculation was performed for the size of this subset. The second observer started from the original images and independently performed cardiac-phase selection, short-axis planning and reformatting where applicable, as well as identification of basal and apical limits and contour generation and correction. No saved analyses or contours from the first observer were reused. Both observers were blinded to the results of the other modality and to each other’s measurements. Representative paired CCT and CMR segmentation and short-axis planning are shown in Figure 1.

In this subset, with O1 and O2 denoting observer 1 and observer 2, three paired differences were defined for each parameter: D_modality_ = CCT_O1_ − CMR_O1_, D_CCTobs_ = CCT_O1_ − CCT_O2_, and D_CMRobs_ = CMR_O1_ − CMR_O2_.

Secondary analyses used LV and RV EDV indexed to BSA (LV EDVi and RV EDVi) and LV mass indexed to BSA, calculated separately at each examination from the height and weight recorded at that time using the Mosteller equation [37]. CCT measurements were therefore indexed to CCT BSA and CMR measurements to CMR BSA. Because raw and indexed variables have different units, whether indexation reduced disagreement was evaluated primarily using dimensionless relative disagreement rather than by directly comparing raw and indexed LoA widths.

Relative cross-modality disagreement was defined as 100 × (CCT − CMR)/[(CCT + CMR)/2], and absolute relative disagreement as its absolute value. This scale was used to compare disagreement across a cohort spanning a wide range of body and cardiac sizes.

End-systolic volumes (ESVs) were analyzed exploratorily in the patients in whom they could be measured. End-systolic CCT reconstructions were generated at the time of the clinical examination and archived in 17 of the 54 patients; in the remaining patients, they could not be produced retrospectively because the CCT raw data were no longer available. In these 17 patients, the primary observer measured LV and RV ESV in both modalities following the same analysis conventions, with end-systole taken as the phase in which the ventricular cavity was smallest, and EF was calculated as 100 × (EDV − ESV)/EDV. Second-observer end-systolic measurements were available for 7 patients of the repeat-measurement subset.

### 2.4. Statistical Analysis

Analyses distinguished agreement, interobserver reproducibility, and reliability in accordance with quantitative-imaging and Guidelines for Reporting Reliability and Agreement Studies (GRRAS) guidance [1,2,3,5]. Throughout the manuscript, “interobserver reproducibility” denotes the within-modality comparison of two independent observers (O1 versus O2, separately for CCT and for CMR), and “cross-modality agreement” denotes the CCT versus CMR comparison; the dispersion of the paired differences in each comparison is referred to as interobserver and cross-modality variability, respectively. Continuous data are reported as the mean ± standard deviation (SD) or median [interquartile range (IQR)], and categorical data are represented as *n* (%). Paired comparisons included mean bias, mean absolute error (MAE), the SD of paired differences [SD(D)], and Bland–Altman 95% LoA; LoA width was calculated as the upper minus the lower LoA [4,38]. Cross-modality concordance was assessed using Lin’s concordance correlation coefficient (CCC) [39], and interobserver reliability was assessed using a two-way random-effects, absolute agreement, single-measure intraclass correlation coefficient (ICC), denoted as ICC(A,1) [40]; absolute agreement was reported, as recommended, and the formula is the same for the two-way random- and mixed-effects forms [41]. Subject-level bootstrap resampling (10,000 iterations) provided 95% confidence intervals (CIs).

The primary variability metric was the SD of paired differences [SD(D)] because it describes dispersion independently of constant bias and determines LoA width. In subjects with complete repeat measurements, ratios were SD(D_modality_)/SD(D_CCTobs_) and SD(D_modality_)/SD(D_CMRobs_). For within-modality observer comparisons, the observer reproducibility coefficient (RC) was defined as 1.96 × SD(D) and was also reported as an interpretable error scale; changing observers technically represents reproducibility rather than strict same-condition repeatability [6]. Variability-ratio CIs used 10,000 paired subject-level bootstrap resamples preserving all comparisons within each patient. Two sensitivity analyses of this comparison were performed in the same subjects. First, the cross-modality comparison was repeated using the second observer’s measurements (CCT O2 versus CMR O2). Second, the three paired differences were expressed on the relative scale, as a percentage of the corresponding pair mean, and the SDs and variability ratios were recalculated.

MAE provided a complementary absolute-error measure. Within the same patients, absolute CCT-CMR differences were compared with CCT and CMR interobserver differences using Wilcoxon signed-rank tests. Effect sizes and bootstrap CIs were emphasized because of the small subset; Benjamini–Hochberg false discovery rate (FDR) adjustment covered the six supportive tests.

Full-cohort cross-modality behavior was additionally described with Bland–Altman analysis, CCC, and Deming regression of CMR on CCT (error-variance ratio 1) [42]. The error-variance ratio of 1, corresponding to equal measurement-error variances in the two modalities, was an assumption of this secondary analysis, because the two error variances could not be estimated separately without repeated examinations. Proportional bias was tested by linear regression of the paired difference (CCT − CMR) on the pair mean, with FDR adjustment across the three principal parameters; these analyses contextualized, rather than defined, the primary variability comparison. Because differences between two methods may be related to the magnitude of the measurement [4], this dependence was examined in the full cohort by Spearman correlation of the absolute CCT-CMR difference and of the absolute relative CCT-CMR difference with the pair mean, with FDR adjustment across the six tests, and by calculating SD(D) within tertiles of the pair mean.

Temporal sensitivity analyses used CCT-CMR intervals of ≤3, ≤6, and ≤12 months, with Spearman correlation between interval and absolute disagreement. BSA-indexed and relative-disagreement analyses were secondary. Exploratory pediatric (<18 years) versus adult comparisons used Mann–Whitney tests with FDR adjustment across six absolute/relative tests. The cross-modality agreement statistics reported for the full cohort were additionally calculated separately within the pediatric group and within the adult group, with bootstrap resampling performed within each group and proportional-bias *q* values FDR-adjusted across the three principal parameters within each group; these within-group estimates are descriptive and hypothesis-generating. ESV and EF were analyzed with the same bias, MAE, SD(D), LoA, and CCC statistics in the 17 patients with end-systolic measurements and with the same paired differences and variability ratios in the 7 patients with second-observer end-systolic measurements; these analyses are exploratory. All analyses were performed in Python 3.13.5 (Python Software Foundation, Beaverton, OR, USA) using NumPy 2.3.5, SciPy 1.17.0, and Matplotlib 3.10.8. Agreement analyses, Deming regression, ICC estimation, multiplicity adjustment, sensitivity analyses, and bootstrap resampling were implemented in custom Python scripts using standard formulas [38,39,40,42].

## 3. Results

### 3.1. Study Population

The final cohort comprised 54 patients, including 32 males (59.3%) and 22 females (40.7%). Thirty-two patients (59.3%) were younger than 18 years and 22 (40.7%) were adults. The median age at CCT was 16.0 years (IQR 12.25–35.0; range 3–68). The median absolute CCT-CMR interval was 4.48 months (IQR 0.76–12.30; range 0.03–34.53). Twenty-four pairs (44.4%) were obtained within 3 months, 28 (51.9%) within 6 months, and 40 (74.1%) within 12 months (Table 1).

Patient and examination characteristics within each age group and in the repeat-measurement subset are given in Supplementary Table S1. In pediatric patients (*n* = 32), the median age was 14.0 years (IQR 9.0–16.0) and the median absolute CCT-CMR interval was 5.80 months (IQR 1.24–12.57). In adult patients (*n* = 22), the median age was 47.5 years (IQR 28.25–54.75) and the median absolute CCT-CMR interval was 1.08 months (IQR 0.30–11.66). The 18-patient repeat-measurement subset comprised 12 pediatric and 6 adult patients, with a median age of 16.0 years (IQR 10.5–23.25; range 3–52) and a median absolute CCT-CMR interval of 1.72 months (IQR 0.86–8.53; range 0.16–12.98). Measurement ranges in this subset were 31–366 mL on CCT and 39–287 mL on CMR for LV EDV, 33–317 mL and 40–267 mL for RV EDV, and 13–213 g and 16–163 g for LV mass; the corresponding full-cohort ranges were 31–366 mL and 39–349 mL, 33–317 mL and 40–340 mL, and 13–245 g and 16–242 g.

### 3.2. Cross-Modality Agreement

**Figure 2:**
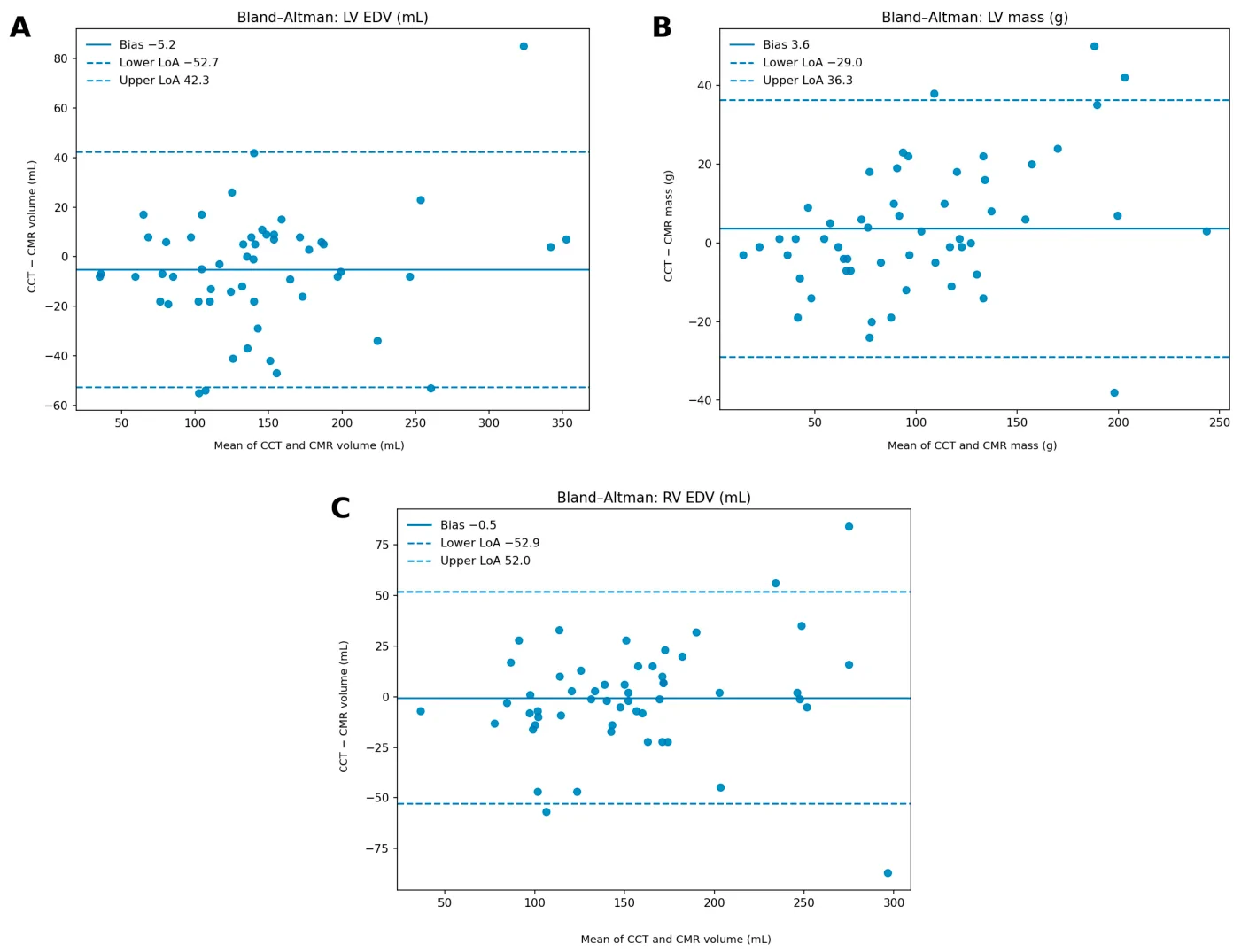
Bland–Altman analysis of full-cohort cross-modality disagreement: (**A**) LV EDV, (**B**) LV myocardial mass, and (**C**) RV EDV. Differences are plotted as CCT minus CMR against the mean of the two measurements. Solid lines indicate mean bias, and dashed lines indicate the 95% limits of agreement; the same sign convention applies to all panels. CCT, cardiac computed tomography; CMR, cardiovascular magnetic resonance; EDV, end-diastolic volume; LoA, limits of agreement; LV, left ventricular; RV, right ventricular.

Full-cohort cross-modality differences had a small mean bias but broad dispersion (Table 2). Figure 2 shows the corresponding Bland–Altman plots for LV EDV, LV myocardial mass, and RV EDV. For LV EDV, CCT and CMR means were 143.50 ± 72.12 mL and 148.72 ± 66.87 mL, respectively, with a bias of −5.22 mL, MAE of 17.59 mL, SD(D) of 24.22 mL, LoA of −52.70 to 42.25 mL, and CCC of 0.937 (95% CI 0.883–0.967). Corresponding values for RV EDV were 154.04 ± 59.77 mL and 154.50 ± 55.30 mL, bias was −0.46 mL, MAE was 18.02 mL, SD(D) was 26.75 mL, LoA was −52.89 to 51.97 mL, and CCC was 0.892 (0.838–0.938). For LV mass, CCT and CMR means were 103.56 ± 54.53 g and 99.93 ± 48.51 g, bias was +3.63 g, MAE was 12.26 g, SD(D) was 16.65 g, LoA was −29.00 to 36.26 g, and CCC was 0.946 (0.914–0.968).

**Figure 3:**
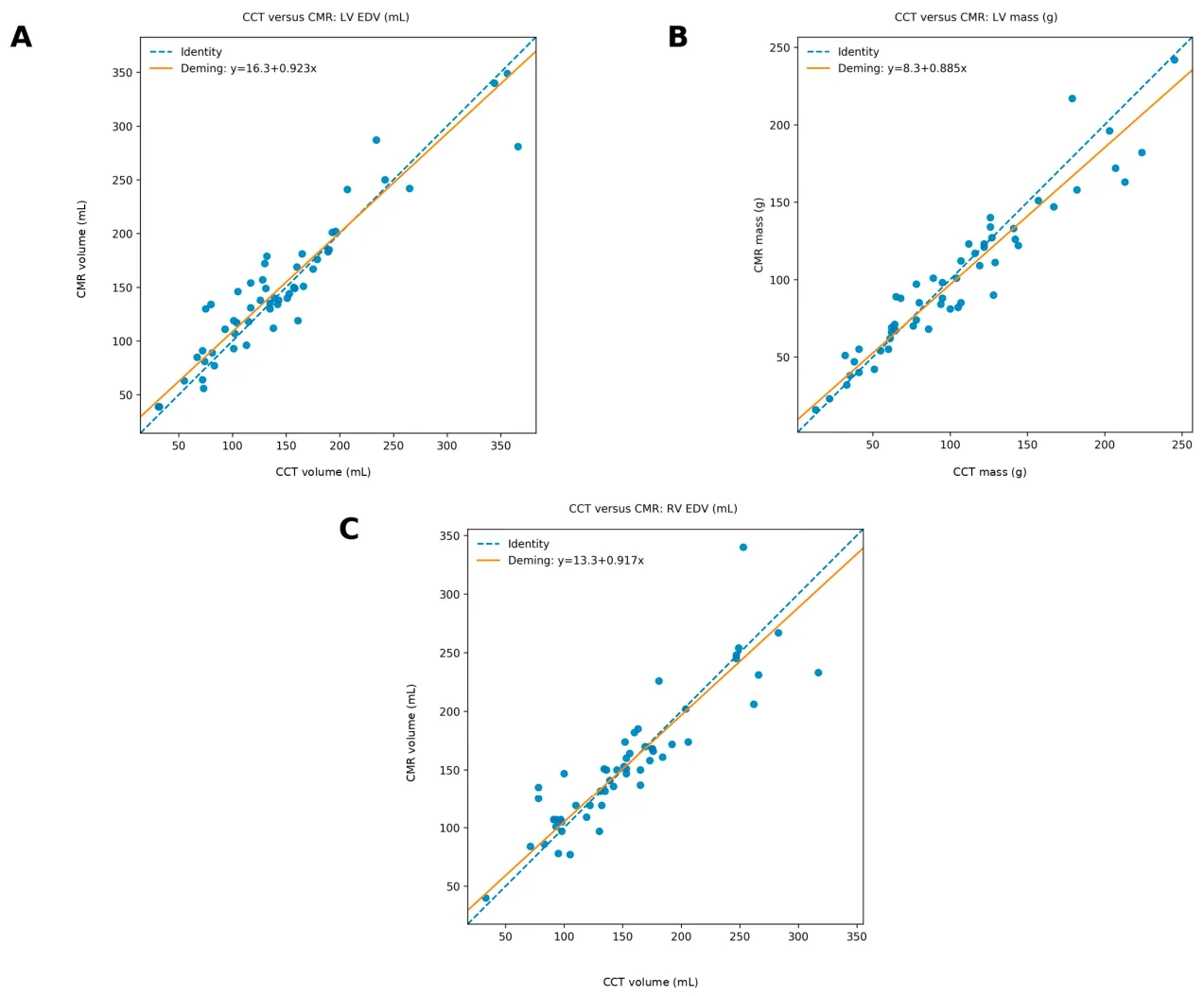
CCT versus CMR for the three primary measurements, with Deming regression of CMR (y axis) on CCT (x axis): (**A**) LV EDV, (**B**) LV myocardial mass, and (**C**) RV EDV. Dashed lines indicate the line of identity and solid lines indicate the fitted Deming relationship. CCT, cardiac computed tomography; CMR, cardiovascular magnetic resonance; EDV, end-diastolic volume; LV, left ventricular; RV, right ventricular.

Mean absolute relative disagreement was 13.52% for LV EDV, 12.12% for RV EDV, and 12.48% for LV mass. Proportional bias remained after FDR correction only for LV mass (*q* = 0.019; Deming slope of CMR on CCT 0.885, 95% CI 0.788–0.992). Deming regression plots are shown in Figure 3. These analyses establish the scale of cross-modality dispersion rather than the primary observer-versus-modality comparison.

The same statistics were calculated separately within each age group (Supplementary Table S2). In pediatric patients (*n* = 32), bias for LV EDV was −10.72 mL, with LoA of −54.28 to 32.84 mL and CCC of 0.904. Corresponding values for RV EDV had a bias of −5.50 mL, LoA of −45.77 to 34.77 mL, and CCC of 0.929. For LV mass, bias was +0.50 g, LoA was −22.05 to 23.05 g, and CCC was 0.975. In adult patients (*n* = 22), bias for LV EDV was +2.77 mL, with LoA of −46.76 to 52.30 mL and CCC of 0.939. Corresponding values for RV EDV were a bias of +6.86 mL, LoA of −57.78 to 71.51 mL and CCC of 0.828. For LV mass, bias was +8.18 g, LoA was −34.20 to 50.57 g, and CCC was 0.855. Within each group, proportional bias was not significant after FDR correction for any parameter. These within-group estimates should be considered hypothesis-generating.

### 3.3. Interobserver Reproducibility

Interobserver variability was substantially smaller than the full-cohort cross-modality dispersion. In the 18-patient repeat-measurement subset, CCT interobserver MAE was 3.67 mL for LV EDV, 4.94 mL for RV EDV, and 5.06 g for LV mass; corresponding RCs were 9.47 mL, 14.36 mL, and 13.16 g. CMR interobserver MAE was 3.28 mL, 2.78 mL, and 4.50 g, with corresponding coefficients of 6.99 mL, 7.02 mL, and 8.05 g, respectively. ICC(A,1) values were 0.990–0.998 for CCT and 0.991–0.999 for CMR. CMR LV mass showed a systematic observer offset of +4.06 g (O1 minus O2) despite ICC(A,1) = 0.991, illustrating why high reliability does not imply negligible absolute observer error.

### 3.4. Comparison of Cross-Modality and Interobserver Variability

The primary comparison used the same 18 patients for LV EDV, RV EDV, and LV mass. In these subjects, the SD of cross-modality differences was 29.78 mL for LV EDV, 29.19 mL for RV EDV, and 16.72 g for LV mass. The corresponding CCT interobserver SDs were 4.83 mL, 7.33 mL, and 6.71 g, while CMR interobserver SDs were 3.57 mL, 3.58 mL, and 4.11 g (Table 3).

**Figure 4:**
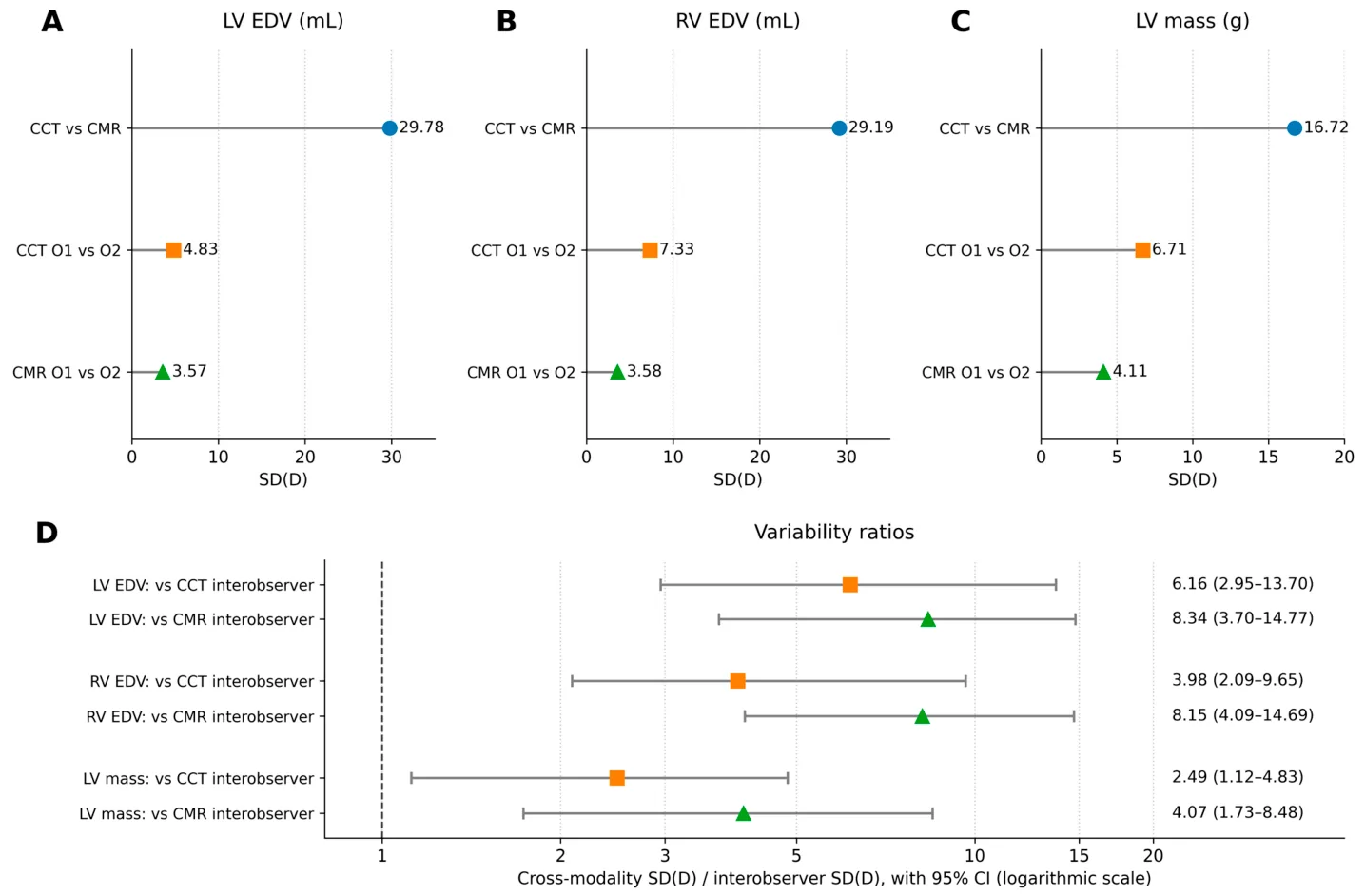
Summary of cross-modality versus interobserver variability in the 18-patient repeat-measurement subset. (**A**–**C**) For each parameter, points show the SD(D) for CCT vs. CMR (blue circles), CCT O1 vs. O2 (orange squares), and CMR O1 vs. O2 (green triangles) comparisons. (**D**) Ratios of cross-modality SD(D) to CCT interobserver SD(D) (orange squares) and to CMR interobserver SD(D) (green triangles), with bootstrap 95% CIs (horizontal bars) on a logarithmic axis; the dashed line indicates a ratio of 1. CCT, cardiac computed tomography; CI, confidence interval; CMR, cardiovascular magnetic resonance; EDV, end-diastolic volume; LV, left ventricular; O1/O2, observer 1/observer 2; RV, right ventricular; SD(D), standard deviation of paired differences.

Cross-modality variability was therefore 6.16-fold higher than CCT interobserver variability for LV EDV (95% CI 2.95–13.70), 3.98-fold higher for RV EDV (2.09–9.65), and 2.49-fold higher for LV mass (1.12–4.83). Relative to CMR interobserver variability, the corresponding ratios were 8.34 (3.70–14.77), 8.15 (4.09–14.69), and 4.07 (1.73–8.48). Because LoA width is proportional to the SD(D), these ratios also describe the relative scale of cross-modality versus interobserver LoA widths. Figure 4 summarizes the cross-modality and interobserver SDs and the corresponding variability ratios.

MAE showed the same pattern. Cross-modality MAE was 20.44 mL for LV EDV, 19.50 mL for RV EDV, and 11.44 g for LV mass, compared with 3.67, 4.94, and 5.06 within CCT and 3.28, 2.78, and 4.50 within CMR. Supportive paired tests of absolute disagreement were directionally consistent; after FDR adjustment, all comparisons remained below 0.05 except LV mass cross-modality versus CCT interobserver disagreement (*q* = 0.052). Given the small subset, the bootstrap effect estimates and CIs were considered more informative than dichotomous significance testing.

Two sensitivity analyses were performed in the same 18 patients (Supplementary Table S3). When the cross-modality comparison was based on the second observer’s measurements (CCT O2 versus CMR O2), SD(D) was 27.76 mL for LV EDV, 32.49 mL for RV EDV, and 14.55 g for LV mass. The corresponding variability ratios were 5.74, 4.44, and 2.17 relative to CCT interobserver variability and 7.78, 9.07, and 3.54 relative to CMR interobserver variability, and all 95% CIs exceeded 1. On the relative scale, the SD of cross-modality differences was 17.63% for LV EDV, 16.96% for RV EDV, and 18.29% for LV mass, compared with 6.52%, 5.40%, and 11.44% within CCT and 3.18%, 2.28%, and 6.87% within CMR. The corresponding ratios were 2.70, 3.14, and 1.60 relative to CCT interobserver variability and 5.54, 7.44, and 2.66 relative to CMR interobserver variability; the 95% CI included 1 only for the LV mass ratio relative to CCT interobserver variability (0.84–3.90).

### 3.5. Examination Interval and Other Determinants of Disagreement

Restricting the analysis to examinations obtained closer in time did not materially reduce cross-modality dispersion (Table 4). For examination intervals of ≤3 months, LoA widths were 101.5 mL for LV EDV, 104.9 mL for RV EDV, and 76.7 g for LV mass; at ≤6 months they were 98.5 mL, 100.8 mL, and 74.2 g; and at ≤12 months they were 98.1 mL, 94.1 mL, and 70.0 g. These values were not consistently narrower than the full-cohort widths of 95.0 mL, 104.9 mL, and 65.3 g. Spearman correlations between examination interval and absolute disagreement were weak and non-significant for LV EDV (ρ = 0.126, *p* = 0.363), RV EDV (ρ = 0.078, *p* = 0.576), and LV mass (ρ = −0.198, *p* = 0.151). These results provide limited evidence that temporal separation alone explains the observed cross-modality dispersion.

BSA indexation also did not materially reduce dimensionless disagreement. Mean absolute relative disagreement was 13.52% for raw LV EDV and 13.58% after indexation, 12.12% versus 12.22% for RV EDV, and 12.48% versus 12.88% for LV mass. Thus, normalization for body size changed the scale of the measurements but did not reduce the relative CCT-CMR discrepancy.

Dependence of cross-modality disagreement on measurement magnitude was examined in the full cohort (Supplementary Table S4). In the lowest, middle, and highest tertiles of the pair mean, SD(D) was 19.79, 22.26, and 30.43 mL for LV EDV; 23.92, 12.37, and 37.36 mL for RV EDV; and 7.01, 17.02, and 20.82 g for LV mass. The absolute difference correlated with the pair mean for LV mass (ρ = 0.384, *q* = 0.012) but not for LV EDV (ρ = −0.001, *q* = 0.992) or RV EDV (ρ = 0.136, *q* = 0.490). The absolute relative difference did not increase with the pair mean and decreased for LV EDV (ρ = −0.402, *q* = 0.012), with no significant correlation for RV EDV (ρ = −0.147, *q* = 0.490) or LV mass (ρ = −0.065, *q* = 0.767).

Exploratory pediatric–adult analyses did not show a robust age-group effect after multiplicity correction. Mean absolute relative LV EDV disagreement was 16.48% in pediatric patients and 9.22% in adults; the unadjusted *p* value was 0.0085, and the FDR-adjusted *q* value was 0.051. RV EDV relative disagreement was similar (12.50% versus 11.57%), and the apparent difference in absolute LV mass MAE (8.69 versus 17.45 g) did not remain significant after FDR correction (*q* = 0.094). These subgroup findings should be considered hypothesis-generating.

### 3.6. End-Systolic Volumes and Ejection Fraction

End-systolic measurements in both modalities were available for 17 patients (10 pediatric patients and seven adults; Supplementary Tables S1 and S5). For LV ESV, bias was +3.29 mL, with LoA of −18.63 to 25.22 mL and CCC of 0.957. Corresponding values for RV ESV were bias of +12.06 mL, LoA of −15.31 to 39.42 mL, and CCC of 0.858. For LV EF, bias was −1.05 percentage points, LoA was −12.84 to 10.75 percentage points, and CCC was 0.733; for RV EF, bias was −3.66 percentage points, LoA was −12.48 to 5.16 percentage points, and CCC was 0.774. In the same patients, mean absolute relative disagreement was 15.43% for LV ESV and 15.47% for RV ESV, compared with 11.32% for LV EDV and 10.85% for RV EDV. In the seven patients with second-observer end-systolic measurements, cross-modality SD(D) was 3.61 and 3.38 times the CCT and CMR interobserver SD(D) for LV ESV and 5.41 and 2.66 times the corresponding interobserver SD(D) for RV ESV. For EF, the corresponding ratios were 1.33 and 1.28 (LV) and 1.77 and 1.05 (RV), and all four 95% CIs included 1. These estimates are exploratory.

## 4. Discussion

### 4.1. Cross-Modality Versus Interobserver Variability

The principal finding is that cross-modality CCT-CMR variability in LV EDV, RV EDV, and LV mass was 2.5- to 8.3-fold greater than interobserver variability measured independently within either modality. In the same repeat-measurement subjects, the dispersion of CCT-CMR differences was approximately 2.5–6.2 times the CCT interobserver dispersion and 4.1–8.3 times the CMR interobserver dispersion, depending on the parameter. The bootstrap CIs for all SD-based variability ratios of the primary analysis remained above 1.0. This changes the interpretation of the full-cohort Bland–Altman results: the broad patient-level CCT-CMR LoAs are not of the same order as ordinary interobserver variability under the study workflow.

This finding did not depend on the observer who performed the cross-modality analysis: with the second observer’s CCT and CMR measurements, the variability ratios were 2.17–5.74 relative to CCT and 3.54–9.07 relative to CMR interobserver variability, and all CIs remained above 1. On the relative scale the ratios were smaller, 1.60–3.14 and 2.66–7.44, respectively, and the CI for LV mass relative to CCT interobserver variability included 1 (0.84–3.90). The excess of cross-modality over interobserver variability is therefore least certain for LV mass relative to CCT interobserver variability; the comparison also gave the smallest ratio on the absolute scale (2.49; 95% CI 1.12–4.83).

This distinction is important because reliability, repeatability, and agreement answer different questions [1,5,6]. ICC is strongly influenced by between-subject heterogeneity and can remain very high when absolute differences are non-negligible. The CMR LV mass measurements illustrate this directly: ICC(A,1) was 0.991 despite an average observer offset of approximately 4 g. Conversely, the cross-modality subset retained CCC values above 0.90 while its SD(D) and MAE were several-fold larger than the corresponding observer metrics. For quantitative imaging, preserving subject ranking is therefore insufficient evidence that measurements can be substituted numerically [4,38,39].

The CMR LV mass offset of +4.06 g (O1 minus O2; LoA −4.00 to 12.11 g) deserves specific comment, although its source cannot be attributed definitively from aggregate measurements. Both observers applied the same convention, assigning papillary muscles and trabeculae to the cavity, so the offset most plausibly reflects consistent differences in border placement at the compacted myocardium–trabecular interface or at the epicardial boundary, or in the inclusion of the most basal section, possibly compounded by the difference in reader experience; the handling of trabeculae and papillary muscles and observer experience is recognized as a source of variation in CMR volume and mass measurements [10,11]. The CCT LV mass offset had the opposite sign (−2.39 g), which argues against a single, modality-independent reader tendency. The CMR offset nevertheless remained small relative to the cross-modality dispersion of LV mass in the same patients (SD(D) 16.72 g).

The direct within-subject comparison provides more information than placing a high interobserver ICC next to a separate CCT-CMR CCC. For LV EDV, cross-modality MAE was 20.4 mL compared with 3.7 mL within CCT and 3.3 mL within CMR. For RV EDV, the corresponding values were 19.5, 4.9, and 2.8 mL; for LV mass, the values were 11.4, 5.1, and 4.5 g. The SD-based ratios were chosen as the primary variability comparison because SD(D) determines LoA width and is not inflated by a constant mean offset. MAE provided a complementary patient-level error scale and showed the same qualitative pattern. Formal *p* values were deliberately secondary because the interobserver subset was small and the variability distributions were not expected to be Gaussian.

Prior CCT-CMR studies established population-level agreement [16,19] and identified technical and phase-related determinants of CCT-CMR differences [19,23,24]. Here, the measurement question is different: is cross-modality dispersion compatible with ordinary interobserver variation measured in the same subjects? This follows quantitative-imaging frameworks that separate agreement from repeatability and reproducibility [1,2,3]; the paired second-observer data provide the internal reference for interobserver reproducibility.

### 4.2. Technical and Image-Related Determinants

The results should not be interpreted as estimating an intrinsic “modality error”. The cross-modality difference necessarily contains observer-dependent measurement errors from both modalities in addition to modality-, acquisition-, physiological-, and time-related components. Accordingly, the variability ratios are comparisons with an empirical intra-modality reproducibility benchmark, not a decomposition or estimate of a pure modality-specific variance component. In a retrospective dataset, the residual CCT-CMR difference can additionally reflect modality-specific contrast and spatial/temporal resolution, cardiac-phase definition, reconstruction, contour visibility, physiological loading conditions, growth or remodeling between examinations, and residual observer effects. Standardized CMR acquisition and post-processing [7,8] reduce but do not eliminate such variation [10,11,12]. Comparisons of CCT with CMR likewise show that disagreement depends on the ventricular parameter [18,19,23], on technical implementation [19], and on the cardiac phase [24]; within CCT, mean volumes also differ between the mid-diastole and end-diastole [22]. The present analysis can therefore localize the scale of interobserver variability relative to the total cross-modality difference, but the design cannot partition cross-modality disagreement into separate contributions from cardiac-phase selection, temporal resolution, heart rate, loading conditions, biological change, and modality-specific image characteristics. Accordingly, the observed CCT-CMR variability should be interpreted as the aggregate disagreement encountered under routine clinical conditions rather than as an estimate of intrinsic modality-specific error.

Image-acquisition factors are a plausible contributor to this residual difference. CCT data were acquired on three scanner platforms with individualized exposure and contrast settings, reflecting routine clinical workflows rather than a harmonized research protocol, whereas all CMR studies used 1.5 T cine imaging with 8 mm sections and a 2 mm gap. Although native CCT series were reconstructed at 0.6–1.25 mm, volumetric analysis of both modalities was performed on short-axis stacks with identical 8 mm/2 mm geometry, so that differences in through-plane sampling were minimized by design. Through-plane partial-volume averaging at the atrioventricular valve plane and apex is nevertheless intrinsic to this geometry in both modalities, and because the position of the most basal section relative to the valve plane is determined independently in each examination, the inclusion or exclusion of a basal section can differ between CCT and CMR. Consistent with this mechanism, basal and inferior RV contours have been identified as the main regional sources of CCT-CMR volume differences, together with differences in respiratory phase at acquisition and contrast-related preload [23]. Differences in spatial resolution, blood–myocardium contrast, and temporal sampling may further affect border definition and end-diastolic phase identification [7,23].

Heart rate, temporal sampling, and the ECG reference used to define the cardiac phase may also contribute to cross-modality disagreement. In congenital heart disease, the CCT-CMR difference in indexed RV ESV increased with heart rate, whereas the differences in the other ventricular parameters did not, and reconstruction of CCT at 5% rather than 10% intervals of the R–R interval did not materially change ventricular volumes at heart rates of up to approximately 80 beats per minute [23]. In a meta-analysis of RV measurements, agreement with CMR did not differ between CCT reconstruction intervals of 5% and 10% of the R–R interval, although a difference in the timing of the end-diastolic phase between the two modalities was proposed as one source of volume differences [19]. These data suggest a limited effect of temporal sampling on EDV at lower heart rates, but they leave the effect at higher heart rates unresolved [23]. In the present cohort, the end-diastole was identified from the images in both modalities, and heart rate, blood pressure, ECG trigger timing, and the increment between reconstructed CCT phases were not consistently available; their contribution, and that of an R-wave- or Q-wave-referenced definition of the end-diastole, therefore, could not be quantified.

The wider scatter at larger volumes in Figure 3 is consistent with the tertile analysis: in absolute units, cross-modality dispersion was greatest in the highest tertile of each parameter, although a significant monotonic association between the absolute difference and magnitude was present only for LV mass. A relationship between difference and magnitude is a recognized feature of method-comparison data [4] and is plausible on geometric grounds, because a given displacement of a contour, or the inclusion or exclusion of one basal section, corresponds to a larger absolute volume or mass in a larger ventricle. A single pair of absolute LoA may therefore understate disagreement in the largest ventricles. On the relative scale, disagreement did not increase with magnitude and, for LV EDV, was larger in smaller ventricles. Absolute and relative measures are therefore complementary in a cohort with this range of cardiac sizes, and the relative-scale analysis of the primary comparison supported the same conclusion as the absolute-scale analysis, with the reservation for LV mass noted above.

The exploratory end-systolic analysis is in line with these observations. Mean absolute relative cross-modality disagreement was larger for ESV than for EDV in the same patients, and CCT gave a larger RV ESV and lower RV EF than CMR, which is the direction reported in congenital heart disease [23]. For EF, which is a ratio of two volumes from the same examination, cross-modality SD(D) was of the same order as interobserver SD(D) in the seven patients with repeated end-systolic analysis, whereas for ESV it was 2.66 to 5.41 times the interobserver SD(D). The excess of cross-modality over interobserver variability shown for EDV and LV mass should therefore not be extrapolated to EF. With 17 and seven patients, these observations require confirmation.

### 4.3. Temporal and Biological Factors

Temporal sensitivity analyses address the time-related component of cross-modality disagreement. If biological change over time was the dominant explanation, substantially narrower dispersion would be expected when CCT and CMR were performed only a few months apart. Instead, LoA widths and CCC values were broadly similar in the subsets with intervals of ≤3, ≤6, and ≤12 months, and the examination interval showed only weak associations with absolute disagreement. This does not establish temporal invariance, particularly in growing children or patients with changing loading conditions. It does, however, provide limited evidence that temporal separation alone explains the observed cross-modality dispersion. The caveat regarding growing children is particularly relevant here, because 59.3% of the cohort was younger than 18 years and the longest interval reached 34.5 months. Ventricular volumes increase with age and body size in children [9,43], so somatic growth alone can change ventricular size over such a period, and structural remodeling may add to this, even when medical and surgical management remain stable. The interval-restricted analyses therefore mitigate, but cannot exclude, a contribution of true biological change, particularly in pediatric patients.

The exploratory age analysis underscores the value of relative error metrics in a mixed pediatric–adult cohort. Absolute differences in milliliters or grams have different implications in a small child and a large adult. Relative LV EDV disagreement was numerically higher in pediatric patients, but the age-group result was borderline after FDR correction, and no consistent pattern was seen for RV EDV or LV mass. The study was not powered for age-specific inference, and incomplete diagnostic characterization precluded meaningful disease-stratified modeling. These findings are best used to motivate larger studies in which age, ventricular size, diagnosis, and acquisition parameters can be modeled jointly.

Body-size normalization is a related consideration in such a cohort. The reference CMR literature demonstrates major age- and body-size effects on ventricular measurements [9,43], as well as sex-related differences in normalized ventricular values [44,45,46]. In the current study, indexation used the BSA recorded at each respective examination, consistent with the source dataset. Mean absolute relative disagreement was essentially unchanged after indexation for all three parameters. Indexation therefore modified the numerical scale of the measurements without mitigating their relative dispersion. This is methodologically plausible: BSA normalization can reduce physiological scale differences across patients but does not correct technical differences in cardiac phase, contrast, reconstruction, or border definition. Indexation should therefore not be expected to make two imaging methods interchangeable.

### 4.4. Clinical Implications

For longitudinal quantitative decisions, we consider consistent modality selection advisable; this extends the established principle that acquisition and measurement methods should remain consistent for serial evaluation [7] to the choice of modality. Because cross-modality variability exceeded interobserver variability by 2.5- to 8.3-fold, a CCT-CMR difference exceeding the expected interobserver variability should not be dismissed as observer-related noise, but it also cannot automatically be labeled biological change. CMR-derived quantitative thresholds should therefore not be applied interchangeably to CCT measurements without accounting for this additional cross-modality uncertainty [23]. Same-modality interstudy variability was not measured in the present study, so this recommendation rests on the magnitude of cross-modality disagreement relative to interobserver variability rather than on a direct comparison of serial CCT-CCT or CMR-CMR examinations. CCT and CMR remain complementary [27,28,29,30]; the present results favor consistency rather than mechanical substitution of numerical thresholds.

### 4.5. Limitations

Limitations include the retrospective, non-simultaneous examinations and absence of a single prospectively standardized protocol, and a heterogeneous clinical composition with incomplete diagnostic labels and examination indications, which precluded diagnostic and indication-specific stratification and may have introduced unmeasured residual variance across anatomical conditions. The primary comparison relied on an 18-patient repeat-measurement subset for which no a priori power calculation was performed. Bootstrap intervals are consequently wide (e.g., 1.12–4.83 for the LV mass ratio relative to CCT interobserver variability), although the lower bounds of all variability ratios of the primary comparison remained above 1.0 on the absolute scale; on the relative scale, the interval for the LV mass ratio relative to CCT interobserver variability included 1 (0.84–3.90). In addition, the interobserver benchmark may not fully represent the broader cohort. The subset had shorter examination intervals than the full cohort (median 1.72 versus 4.48 months) and, with 12 pediatric and six adult patients, was too small for age-specific estimates of the variability ratios. Interobserver variability was measured on one platform under shared analysis conventions and therefore does not capture cross-platform, cross-center, or algorithm-related reproducibility [47]. Modality effects cannot be separated definitively from phase, acquisition, or physiological differences, and the intrinsic modality error was not estimated. The examination interval reached 34.5 months in a cohort in which most patients were children, so somatic growth may have contributed to cross-modality differences despite the interval-restricted sensitivity analyses. The source of the systematic CMR LV mass offset between observers could not be resolved from aggregate measurements. Pediatric–adult analyses were exploratory and unadjusted for diagnosis or clinical state.

Heart rate and blood pressure during the examinations, ECG trigger timing, and the number and increment of the reconstructed CCT phases were not consistently available in the retrospective source data. The analyses were performed on the archived image series, in which heart rate, trigger timing, and the increment between reconstructed phases were not consistently recorded; the CCT raw data were no longer available. The potential contribution of these factors to cross-modality variability therefore could not be quantified. Alternative R-wave- and Q-wave-referenced reconstructions were likewise not available, so the effect of the ECG reference on the measured volumes could not be examined. ESV and EF could be analyzed only exploratorily, in the 17 patients with archived end-systolic CCT reconstructions and, for the interobserver comparison, in seven patients; the corresponding estimates, in particular the variability ratios, are imprecise. Whether cross-modality and interobserver variability differ for ESV and EF should be addressed in a dedicated study. Intraobserver variability was not assessed, because no analysis was repeated by the same observer. Differences between two observers include each observer’s own measurement error in addition to any systematic difference between them, so interobserver variability is the more conservative benchmark for the present comparison, but the within-observer component could not be reported separately.

## 5. Conclusions

In this heterogeneous pediatric–adult cohort examined under routine clinical conditions, cross-modality CCT-CMR disagreement in LV EDV, RV EDV, and LV mass was 2.5- to 8.3-fold larger than interobserver variability within either modality. The findings suggest that clinically relevant CCT-CMR differences are unlikely to be explained primarily by observer-related post-processing variability and are more likely to reflect a combination of modality-specific acquisition, reconstruction, cardiac-phase, and physiological factors that the present design cannot separate. For longitudinal quantitative assessment, consistent selection of imaging modality and an analysis approach appears preferable to direct interchange of quantitative thresholds between CCT and CMR.

## Figures and Tables

**Table 1:** Patient and examination characteristics.

Characteristic	Value
Patients, *n*	54
Male sex, *n* (%)	32 (59.3%)
Pediatric patients (<18 years), *n* (%)	32 (59.3%)
Adult patients (≥18 years), *n* (%)	22 (40.7%)
Age at CCT, years	16.0 [12.25–35.0]
Age range, years	3–68
Absolute CCT-CMR interval, months	4.48 [0.76–12.30]
Interval ≤ 3 months, *n* (%)	24 (44.4%)
Interval ≤ 6 months, *n* (%)	28 (51.9%)
Interval ≤ 12 months, *n* (%)	40 (74.1%)

Continuous variables are the median [IQR] unless otherwise stated. CCT, cardiac computed tomography; CMR, cardiovascular magnetic resonance; IQR, interquartile range.

**Table 2:** Cross-modality CCT-CMR agreement in the full cohort.

Parameter	CCT Mean ± SD	CMR Mean ± SD	Bias	MAE	SD(D)	95% LoA	CCC (95% CI)	Prop. Bias *q*
LV EDV, mL	143.50 ± 72.12	148.72 ± 66.87	−5.22	17.59	24.22	−52.70 to 42.25	0.937 (0.883–0.967)	0.165
RV EDV, mL	154.04 ± 59.77	154.50 ± 55.30	−0.46	18.02	26.75	−52.89 to 51.97	0.892 (0.838–0.938)	0.215
LV mass, g	103.56 ± 54.53	99.93 ± 48.51	+3.63	12.26	16.65	−29.00 to 36.26	0.946 (0.914–0.968)	0.019

Bias is CCT minus CMR. Proportional bias *q* values are Benjamini–Hochberg-adjusted across the three principal parameters. CCC, Lin concordance correlation coefficient; CCT, cardiac computed tomography; CI, confidence interval; CMR, cardiovascular magnetic resonance; EDV, end-diastolic volume; LoA, limits of agreement; LV, left ventricular; MAE, mean absolute error; *q*, false discovery rate-adjusted *p* value; RV, right ventricular; SD, standard deviation; SD(D), standard deviation of paired differences.

**Table 3:** Primary comparison of cross-modality and interobserver variability in the same subjects.

Measure	Pair	*n*	Bias	MAE	SD(D)	95% LoA	LoA Width	CCC/ICC (95% CI)	SD Ratio *
LV EDV, mL	CCT vs. CMR	18	−5.56	20.44	29.78	−63.92 to 52.81	116.73	CCC 0.925 (0.859–0.966)	6.16/8.34
LV EDV, mL	CCT O1 vs. O2	18	+0.78	3.67	4.83	−8.70 to 10.25	18.95	ICC 0.998 (0.994–0.999)	—
LV EDV, mL	CMR O1 vs. O2	18	−1.50	3.28	3.57	−8.49 to 5.49	13.98	ICC 0.999 (0.995–0.999)	—
RV EDV, mL	CCT vs. CMR	18	+1.06	19.50	29.19	−56.16 to 58.28	114.44	CCC 0.920 (0.827–0.976)	3.98/8.15
RV EDV, mL	CCT O1 vs. O2	18	+1.83	4.94	7.33	−12.53 to 16.19	28.72	ICC 0.996 (0.984–0.999)	—
RV EDV, mL	CMR O1 vs. O2	18	−0.33	2.78	3.58	−7.35 to 6.69	14.04	ICC 0.999 (0.996–0.999)	—
LV mass, g	CCT vs. CMR	18	−0.56	11.44	16.72	−33.33 to 32.22	65.55	CCC 0.934 (0.877–0.975)	2.49/4.07
LV mass, g	CCT O1 vs. O2	18	−2.39	5.06	6.71	−15.55 to 10.77	26.32	ICC 0.990 (0.969–0.997)	—
LV mass, g	CMR O1 vs. O2	18	+4.06	4.50	4.11	−4.00 to 12.11	16.11	ICC 0.991 (0.973–0.997)	—

* For CCT-vs-CMR rows, the variability ratio is the SD of cross-modality differences divided by the CCT interobserver SD/CMR interobserver SD. Bootstrap 95% CIs: LV EDV 2.95–13.70/3.70–14.77; RV EDV 2.09–9.65/4.09–14.69; LV mass 1.12–4.83/1.73–8.48. Within-modality observer reproducibility coefficients [1.96 × SD(D)] were 9.47/6.99 mL for CCT/CMR LV EDV, 14.36/7.02 mL for RV EDV, and 13.16/8.05 g for LV mass. CCC, Lin concordance correlation coefficient; CCT, cardiac computed tomography; CI, confidence interval; CMR, cardiovascular magnetic resonance; EDV, end-diastolic volume; ICC, intraclass correlation coefficient; LoA, limits of agreement; LV, left ventricular; MAE, mean absolute error; O1/O2, observer 1/observer 2; RV, right ventricular; SD(D), standard deviation of paired differences.

**Table 4:** Secondary sensitivity and determinant analyses.

Panel	Analysis	LV EDV	RV EDV	LV Mass
A	≤3 months (*n* = 24): LoA width/CCC	101.5/0.953	104.9/0.904	76.7/0.906
A	≤6 months (*n* = 28): LoA width/CCC	98.5/0.951	100.8/0.901	74.2/0.912
A	≤12 months (*n* = 40): LoA width/CCC	98.1/0.943	94.1/0.904	70.0/0.942
A	Interval vs. absolute CCT-CMR difference: Spearman ρ (*p*)	0.126 (0.363)	0.078 (0.576)	−0.198 (0.151)
B	Mean absolute relative disagreement: raw	13.52%	12.12%	12.48%
B	Mean absolute relative disagreement: indexed	13.58%	12.22%	12.88%
C	Pediatric mean absolute relative disagreement	16.48%	12.50%	12.15%
C	Adult mean absolute relative disagreement	9.22%	11.57%	12.96%
C	Age-group FDR *q* for relative disagreement	0.051	0.799	0.799

Panel A: Examination-interval sensitivity analyses; LoA width is upper minus lower LoA. Panel B: BSA indexation used examination-specific CCT and CMR BSA values. Panel C: Pediatric versus adult comparison is exploratory; *q* values are Benjamini–Hochberg-adjusted across six age-group tests (absolute and relative disagreement for three parameters). BSA, body surface area; CCC, Lin concordance correlation coefficient; CCT, cardiac computed tomography; CMR, cardiovascular magnetic resonance; EDV, end-diastolic volume; FDR, false discovery rate; LoA, limits of agreement; LV, left ventricular; RV, right ventricular; ρ, Spearman rank correlation coefficient.

## Data Availability

The data supporting this study are available from the corresponding authors upon reasonable request, pending institutional privacy approvals.

## Supplementary Materials

The following supporting information can be downloaded at https://www.mdpi.com/article/10.3390/diagnostics16203280/s1. Table S1: Patient and examination characteristics of the full cohort, of the pediatric and adult groups, and of the repeat-measurement and end-systolic subsets; Table S2: Age-stratified cross-modality CCT-CMR agreement in pediatric and adult patients; Table S3: Sensitivity analyses of the primary comparison of cross-modality and interobserver variability in the 18-patient repeat-measurement subset; Table S4: Dependence of cross-modality CCT-CMR disagreement on measurement magnitude in the full cohort; Table S5: Exploratory analysis of end-systolic volumes and ejection fraction.

## Abbreviations

Abbreviation	Definition
BSA	Body surface area
CCC	Lin concordance correlation coefficient
CCT	Cardiac computed tomography
CI	Confidence interval
CMR	Cardiovascular magnetic resonance
ECG	Electrocardiogram
EDV	End-diastolic volume
EDVi	End-diastolic volume indexed to body surface area
EF	Ejection fraction
ESV	End-systolic volume
FDR	False discovery rate (Benjamini–Hochberg adjustment)
GRRAS	Guidelines for Reporting Reliability and Agreement Studies
ICC	Intraclass correlation coefficient
IQR	Interquartile range
LoA	Limits of agreement
LV	Left ventricular
MAE	Mean absolute error
O1/O2	Observer 1/Observer 2
RC	Observer-reproducibility coefficient [1.96 × SD(D)]
RV	Right ventricular
SD	Standard deviation
SD(D)	Standard deviation of paired differences
