# Supplementary material for: Observer-Related Versus Cross-Modality Variability in Ventricular Quantification by Cardiac CT and Cardiovascular Magnetic Resonance
Source: Diagnostics (Basel). 2026 Oct 9;16(20):3280. doi: 10.3390/diagnostics16203280 (PMC13703353; doi:10.3390/diagnostics16203280)
Supplement: Supplementary file 1 [file diagnostics-16-03280-s001.zip › diagnostics-4604437-supplementary.pdf]

Supplementary Materials

Observer-Related Versus Cross-Modality Variability in Ventricular Quantification by Cardiac CT and Cardiovascular Magnetic Resonance

Răzvan-Andrei Licu, Alexandra-Mihaela Țolescu-Dobra, Gheorghe-Marian Rotariu, Kincső-Zsófia Lőrincz, Claudia-Raluca Mariean, Marian Pop, Dragoș Constantin Cucoranu, Liviu Moraru, Andrei Manea, Dariana-Elena Pătrîntașu, Anca Dumitrașcu and Anca Bacârea

Table S1. Patient and examination characteristics of the full cohort, of the pediatric and adult groups, and of the repeat-measurement and end-systolic subsets.

Characteristic	Full cohort (*n* = 54)	Pediatric patients, <18 years (*n* = 32)	Adult patients, ≥18 years (*n* = 22)	Repeat-measurement subset (*n* = 18)	End-systolic subset (*n* = 17)
Male sex, *n* (%)	32 (59.3%)	20 (62.5%)	12 (54.5%)	9 (50.0%)	13 (76.5%)
Pediatric patients (<18 years), *n* (%)	32 (59.3%)	—	—	12 (66.7%)	10 (58.8%)
Adult patients (≥18 years), *n* (%)	22 (40.7%)	—	—	6 (33.3%)	7 (41.2%)
Age, years	16.0 [12.25–35.0]	14.0 [9.0–16.0]	47.5 [28.25–54.75]	16.0 [10.5–23.25]	17.0 [15.0–25.0]
Age range, years	3–68	3–17	19–68	3–52	3–68
Absolute CCT-CMR interval, months	4.48 [0.76–12.30]	5.80 [1.24–12.57]	1.08 [0.30–11.66]	1.72 [0.86–8.53]	1.22 [0.53–8.84]
Interval range, months	0.03–34.53	0.03–34.53	0.03–32.53	0.16–12.98	0.03–22.11
Interval ≤3 months, *n* (%)	24 (44.4%)	12 (37.5%)	12 (54.5%)	11 (61.1%)	10 (58.8%)
Interval ≤6 months, *n* (%)	28 (51.9%)	16 (50.0%)	12 (54.5%)	12 (66.7%)	10 (58.8%)
Interval ≤12 months, *n* (%)	40 (74.1%)	23 (71.9%)	17 (77.3%)	17 (94.4%)	14 (82.4%)
LV EDV range, mL (CCT / CMR)	31–366 / 39–349	31–242 / 39–287	101–366 / 118–349	31–366 / 39–287	31–344 / 39–340
RV EDV range, mL (CCT / CMR)	33–317 / 40–340	33–266 / 40–254	93–317 / 97–340	33–317 / 40–267	33–266 / 40–245
LV mass range, g (CCT / CMR)	13–245 / 16–242	13–245 / 16–242	62–224 / 69–217	13–213 / 16–163	22–245 / 23–242

Continuous variables are median [IQR] unless otherwise stated; ranges are minimum–maximum. The full-cohort column is given for reference (see Table 1 of the main text). The repeat-measurement subset comprises the 18 patients in whom the second observer independently repeated the complete CCT and CMR analysis (Table 3 of the main text). The end-systolic subset comprises the 17 patients with end-systolic measurements in both modalities (Table S5). Characteristics are reported descriptively within each group. CCT, cardiac computed tomography; CMR, cardiovascular magnetic resonance; EDV, end-diastolic volume; IQR, interquartile range; LV, left ventricular; RV, right ventricular.

Table S2. Age-stratified cross-modality CCT-CMR agreement in pediatric and adult patients.

Parameter	CCT mean ± SD	CMR mean ± SD	Bias	MAE	SD(D)	95% LoA	CCC (95% CI)	Prop. bias *q*
**Pediatric patients (<18 years), *n* = 32**								
LV EDV, mL	116.06 ± 53.15	126.78 ± 58.50	−10.72	18.59	22.22	−54.28 to 32.84	0.904 (0.802–0.950)	0.264
RV EDV, mL	141.41 ± 57.85	146.91 ± 54.77	−5.50	15.50	20.55	−45.77 to 34.77	0.929 (0.854–0.967)	0.405
LV mass, g	85.69 ± 53.30	85.19 ± 49.63	+0.50	8.69	11.50	−22.05 to 23.05	0.975 (0.932–0.989)	0.220
**Adult patients (≥18 years), *n* = 22**								
LV EDV, mL	183.41 ± 78.38	180.64 ± 66.58	+2.77	16.14	25.27	−46.76 to 52.30	0.939 (0.836–0.985)	0.079
RV EDV, mL	172.41 ± 58.97	165.55 ± 55.44	+6.86	21.68	32.98	−57.78 to 71.51	0.828 (0.738–0.913)	0.620
LV mass, g	129.55 ± 46.03	121.36 ± 38.62	+8.18	17.45	21.63	−34.20 to 50.57	0.855 (0.773–0.903)	0.160

Statistics correspond to those reported for the full cohort in Table 2 of the main text and were calculated separately within each age group. Bias is CCT minus CMR. The 95% CIs for CCC were obtained by subject-level bootstrap resampling (10,000 iterations) within each age group. Proportional bias *q* values are Benjamini–Hochberg adjusted across the three principal parameters within each age group. These subgroup analyses are exploratory and hypothesis-generating; the study was not powered for age-specific inference. CCC, Lin concordance correlation coefficient; CCT, cardiac computed tomography; CI, confidence interval; CMR, cardiovascular magnetic resonance; EDV, end-diastolic volume; LoA, limits of agreement; LV, left ventricular; MAE, mean absolute error; *q*, false discovery rate-adjusted *p* value; RV, right ventricular; SD, standard deviation; SD(D), standard deviation of paired differences.

Table S3. Sensitivity analyses of the primary comparison of cross-modality and interobserver variability in the 18-patient repeat-measurement subset.

Panel A. Cross-modality comparison based on the second observer's measurements

Measure	Cross-modality comparison	Bias	MAE	SD(D)	95% LoA	CCC (95% CI)	SD ratio vs. CCT interobserver (95% CI)	SD ratio vs. CMR interobserver (95% CI)
LV EDV, mL	CCT O1 vs. CMR O1 (Table 3)	−5.56	20.44	29.78	−63.92 to 52.81	0.925 (0.859–0.966)	6.16 (2.95–13.70)	8.34 (3.70–14.77)
	CCT O2 vs. CMR O2	−7.83	19.72	27.76	−62.23 to 46.57	0.934 (0.845–0.971)	5.74 (2.51–13.27)	7.78 (3.37–13.95)
RV EDV, mL	CCT O1 vs. CMR O1 (Table 3)	+1.06	19.50	29.19	−56.16 to 58.28	0.920 (0.827–0.976)	3.98 (2.09–9.65)	8.15 (4.09–14.69)
	CCT O2 vs. CMR O2	−1.11	22.33	32.49	−64.80 to 62.58	0.901 (0.789–0.970)	4.44 (2.70–10.06)	9.07 (4.71–15.89)
LV mass, g	CCT O1 vs. CMR O1 (Table 3)	−0.56	11.44	16.72	−33.33 to 32.22	0.934 (0.877–0.975)	2.49 (1.12–4.83)	4.07 (1.73–8.48)
	CCT O2 vs. CMR O2	+5.89	10.44	14.55	−22.62 to 34.40	0.942 (0.893–0.981)	2.17 (1.09–4.24)	3.54 (1.66–7.47)

Panel B. Primary comparison on the relative scale

Measure	Pair	Bias, %	MAE, %	SD(D), %	95% LoA, %	SD ratio vs. CCT interobserver (95% CI)	SD ratio vs. CMR interobserver (95% CI)
LV EDV	CCT O1 vs. CMR O1	−9.93	15.83	17.63	−44.48 to 24.63	2.70 (1.30–8.51)	5.54 (3.07–10.59)
	CCT O1 vs. O2	+1.83	3.91	6.52	−10.94 to 14.61	—	—
	CMR O1 vs. O2	−0.69	2.70	3.18	−6.92 to 5.54	—	—
RV EDV	CCT O1 vs. CMR O1	−3.71	12.56	16.96	−36.96 to 29.53	3.14 (1.80–5.90)	7.44 (4.13–12.46)
	CCT O1 vs. O2	+1.37	3.94	5.40	−9.21 to 11.95	—	—
	CMR O1 vs. O2	−0.42	1.87	2.28	−4.89 to 4.05	—	—
LV mass	CCT O1 vs. CMR O1	−5.36	14.46	18.29	−41.21 to 30.50	1.60 (0.84–3.90)	2.66 (1.51–5.04)
	CCT O1 vs. O2	−1.28	7.67	11.44	−23.71 to 21.15	—	—
	CMR O1 vs. O2	+5.35	6.80	6.87	−8.12 to 18.81	—	—

Both panels use the 18 patients of Table 3 of the main text. Panel A: for each measure, the first row repeats the primary cross-modality comparison (first observer) from Table 3 for reference, and the second row gives the same comparison based on the second observer's independent CCT and CMR measurements. Panel B: every paired difference of the primary analysis was expressed as 100 × difference/pair mean, so that bias, MAE, SD(D), and LoA are percentages. In both panels, the variability ratio is the SD of the cross-modality differences divided by the SD of the CCT interobserver differences (CCT O1 vs. O2) and by the SD of the CMR interobserver differences (CMR O1 vs. O2) on the same scale. Bias is CCT minus CMR, or O1 minus O2. The 95% CIs of the sensitivity analyses were obtained from 10,000 paired subject-level bootstrap resamples. CCC, Lin concordance correlation coefficient; CCT, cardiac computed tomography; CI, confidence interval; CMR, cardiovascular magnetic resonance; EDV, end-diastolic volume; LoA, limits of agreement; LV, left ventricular; MAE, mean absolute error; O1/O2, observer 1/observer 2; RV, right ventricular; SD, standard deviation; SD(D), standard deviation of paired differences.

Table S4. Dependence of cross-modality CCT-CMR disagreement on measurement magnitude in the full cohort.

Parameter	Tertile of pair mean	*n*	Pair-mean range	Bias	SD(D)	Mean absolute relative disagreement, %	Absolute difference vs. pair mean: Spearman ϱ (*p*; *q*)	Absolute relative difference vs. pair mean: Spearman ϱ (*p*; *q*)
LV EDV, mL	Lowest	18	35.0–110.5	−10.11	19.79	19.24	−0.001 (0.992; 0.992)	−0.402 (0.003; 0.012)
	Middle	19	116.5–153.5	−3.95	22.26	12.24		
	Highest	17	155.5–352.5	−1.47	30.43	8.88		
RV EDV, mL	Lowest	18	36.5–123.5	−8.11	23.92	18.45	0.136 (0.326; 0.490)	−0.147 (0.288; 0.490)
	Middle	18	125.5–169.5	+0.50	12.37	6.20		
	Highest	18	171.0–296.5	+6.22	37.36	11.71		
LV mass, g	Lowest	18	14.5–76.0	−2.50	7.01	11.85	0.384 (0.004; 0.012)	−0.065 (0.639; 0.767)
	Middle	18	77.0–117.5	+2.78	17.02	15.14		
	Highest	18	120.0–243.5	+10.61	20.82	10.45		

The pair mean is (CCT + CMR)/2. Tertiles were defined by the 33.3rd and 66.7th percentiles of the pair mean; equal pair means were assigned to the same tertile. Pair-mean range, bias, and SD(D) are in the units of the parameter; bias is CCT minus CMR and SD(D) is the SD of the CCT minus CMR differences within the tertile. Relative disagreement is 100 × (CCT − CMR)/pair mean. Spearman correlations relate the absolute difference and the absolute relative difference to the pair mean across all 54 patients; *q* values are Benjamini–Hochberg adjusted across the six tests. CCT, cardiac computed tomography; CMR, cardiovascular magnetic resonance; EDV, end-diastolic volume; LV, left ventricular; *q*, false discovery rate-adjusted *p* value; RV, right ventricular; SD, standard deviation; SD(D), standard deviation of paired differences; ϱ, Spearman rank correlation coefficient.

Table S5. Exploratory analysis of end-systolic volumes and ejection fraction.

Panel A. Cross-modality CCT-CMR agreement in the 17 patients with end-systolic measurements

Parameter	CCT mean ± SD	CMR mean ± SD	Bias	MAE	SD(D)	95% LoA	CCC (95% CI)	Mean absolute relative disagreement, %
LV EDV, mL	161.06 ± 75.22	159.24 ± 78.62	+1.82	16.06	22.10	−41.50 to 45.14	0.958 (0.880–0.987)	11.32
LV ESV, mL	73.00 ± 39.24	69.71 ± 39.95	+3.29	8.00	11.18	−18.63 to 25.22	0.957 (0.861–0.987)	15.43
LV EF, %	55.70 ± 7.14	56.74 ± 9.37	−1.05	4.69	6.02	−12.84 to 10.75	0.733 (0.548–0.846)	—
RV EDV, mL	163.82 ± 63.63	152.71 ± 54.18	+11.12	16.65	19.69	−27.48 to 49.72	0.928 (0.831–0.972)	10.85
RV ESV, mL	89.94 ± 37.31	77.88 ± 29.20	+12.06	13.47	13.96	−15.31 to 39.42	0.858 (0.693–0.942)	15.47
RV EF, %	45.55 ± 8.60	49.21 ± 7.84	−3.66	4.27	4.50	−12.48 to 5.16	0.774 (0.607–0.880)	—

Panel B. Cross-modality and interobserver variability in the 7 patients with second-observer end-systolic measurements

Parameter	Pair	Bias	MAE	SD(D)	95% LoA	SD ratio vs. CCT interobserver (95% CI)	SD ratio vs. CMR interobserver (95% CI)
LV ESV, mL	CCT O1 vs. CMR O1	−1.71	8.86	11.37	−24.00 to 20.57	3.61 (1.81–5.86)	3.38 (1.76–6.85)
	CCT O1 vs. O2	−0.29	2.57	3.15	−6.45 to 5.88	—	—
	CMR O1 vs. O2	−1.57	2.71	3.36	−8.16 to 5.01	—	—
LV EF, %	CCT O1 vs. CMR O1	+0.53	5.04	5.97	−11.17 to 12.23	1.33 (0.75–5.63)	1.28 (0.57–5.83)
	CCT O1 vs. O2	+1.44	3.30	4.48	−7.34 to 10.22	—	—
	CMR O1 vs. O2	+2.22	2.98	4.65	−6.89 to 11.32	—	—
RV ESV, mL	CCT O1 vs. CMR O1	+10.71	14.14	17.11	−22.83 to 44.26	5.41 (3.39–11.52)	2.66 (1.03–13.62)
	CCT O1 vs. O2	−2.00	3.14	3.16	−8.20 to 4.20	—	—
	CMR O1 vs. O2	−2.57	4.00	6.43	−15.17 to 10.02	—	—
RV EF, %	CCT O1 vs. CMR O1	−3.56	5.03	5.54	−14.42 to 7.31	1.77 (0.94–4.92)	1.05 (0.49–4.19)
	CCT O1 vs. O2	+1.88	2.41	3.14	−4.26 to 8.03	—	—
	CMR O1 vs. O2	+0.87	3.35	5.29	−9.50 to 11.23	—	—

Panel A: the 17 patients with end-systolic measurements in both modalities; the EDV rows refer to the same patients. Panel B: the 7 patients of the repeat-measurement subset in whom the second observer also measured end-systolic volumes; variability ratios are defined as in Table S3. EF was calculated as 100 × (EDV − ESV)/EDV; for EF, bias, MAE, SD(D), and LoA are in percentage points, and relative disagreement [100 × (CCT − CMR)/pair mean] was not calculated. Bias is CCT minus CMR, or O1 minus O2. The 95% CIs were obtained from 10,000 subject-level bootstrap resamples; with 7 patients, the intervals of Panel B are approximate. CCC, Lin concordance correlation coefficient; CCT, cardiac computed tomography; CI, confidence interval; CMR, cardiovascular magnetic resonance; EDV, end-diastolic volume; EF, ejection fraction; ESV, end-systolic volume; LoA, limits of agreement; LV, left ventricular; MAE, mean absolute error; O1/O2, observer 1/observer 2; RV, right ventricular; SD, standard deviation; SD(D), standard deviation of paired differences.
